# Optic Nerve Sheath Diameter in Preterm Infants: Relationship with Respiratory Support and the Influence of Gestational Maturity

**DOI:** 10.3390/jcm15103732

**Published:** 2026-05-13

**Authors:** Ozlem Unal, Burak Ceran, Rana Beyoglu, Hayriye Gozde Kanmaz Kutman

**Affiliations:** 1 Department of Radiology, Faculty of Medicine, Ankara Yıldırım Beyazıt University, Ankara 06800, Turkey; 2 Department of Neonatology, Ankara Bilkent City Hospital, University of Health Sciences, Ankara 06800, Turkey; 3 Department of Pediatric Radiology, Ankara Bilkent City Hospital, University of Health Sciences, Ankara 06800, Turkey

**Keywords:** optic nerve sheath diameter, preterm infants, point-of-care ultrasound, gestational age, respiratory support, neonatal intensive care

## Abstract

**Background/Objective:** Bedside ultrasonographic measurement of optic nerve sheath diameter (ONSD) has increasingly been used as a non-invasive method for evaluating intracranial dynamics. In preterm infants, interpretation of these measurements is complicated by the strong influence of gestational maturity. The objective of this study was to examine the relationship between ONSD and respiratory support in preterm infants and to determine whether this relationship reflects an independent physiological effect or is mainly related to maturational confounding. **Methods:** This retrospective single-center study included 110 preterm infants. ONSD measurements were obtained at the bedside using a standardized ultrasonographic technique. Infants were categorized according to the need for invasive mechanical ventilation. Associations between ONSD, respiratory parameters, and clinical variables were evaluated with correlation analyses and multivariable logistic regression after adjustment for gestational age and birth weight. **Results:** ONSD values were lower in infants who required invasive mechanical ventilation and who also had lower gestational age and birth weight. After adjustment for these variables, the association between ONSD and invasive ventilation became less pronounced. Although ONSD showed a moderate unadjusted correlation with SpO_2_, no consistent independent association with respiratory parameters remained after adjustment for maturational factors. The difference in ONSD between groups was small (0.48 mm) and within the expected range of measurement variability. **Conclusions:** In this cohort, differences in ONSD according to respiratory support appeared to be more closely related to maturational status than to respiratory disease severity. ONSD measurements in preterm infants should therefore be interpreted within the clinical context of prematurity rather than used alone as indicators of respiratory status.

## 1. Introduction

Point-of-care ultrasonography is widely used in neonatal intensive care because it provides rapid bedside evaluation without radiation exposure or the need for patient transport [1,2,3]. In recent years, ultrasonographic measurement of optic nerve sheath diameter (ONSD) has attracted increasing interest as a non-invasive marker associated with intracranial dynamics [4,5,6,7,8,9].

Interpreting ONSD measurements in preterm infants remains difficult. Unlike older children and adults, ONSD values in premature neonates are strongly influenced by gestational maturity. Previous neonatal studies have shown close relationships between ONSD, gestational age, birth weight, and other maturational parameters [10,11,12,13,14]. These findings suggest that baseline ONSD measurements in preterm infants are shaped largely by developmental maturity.

Our earlier study also demonstrated strong associations between ONSD measurements and maturational parameters in preterm infants [13]. This raises an important question: do apparent associations between ONSD and respiratory support represent true physiological effects, or are they mainly related to maturational confounding?

This issue is clinically relevant because the need for respiratory support in preterm infants is closely linked to gestational age and overall clinical instability. Mechanical ventilation may affect intracranial dynamics through changes in intrathoracic pressure and cerebral venous return [15,16,17,18,19]. In premature infants, however, these effects may be difficult to separate from the much stronger influence of maturation itself.

The present study was designed as a secondary mechanistic analysis to examine whether the relationship between ONSD and respiratory support remains after adjustment for gestational age and birth weight. Unlike previous studies focused primarily on normative or anthropometric associations, the present analysis specifically examined whether respiratory support was independently associated with ONSD after adjustment for gestational maturity. We hypothesized that any apparent association would become substantially weaker after accounting for gestational and developmental factors.

## 2. Materials and Methods

### 2.1. Study Population and Design

This study was conducted in a tertiary neonatal intensive care unit and was based on a retrospective secondary analysis of a previously established cohort of preterm infants monitored between January 2020 and June 2021. Bedside ultrasonographic optic nerve sheath diameter (ONSD) measurements had originally been obtained during routine clinical care. Some infants included in the present analysis were also part of a previously published cohort investigating maturational and anthropometric associations of ONSD. The current study, however, focused specifically on the relationship between ONSD and respiratory support after adjustment for gestational age and birth weight.

Infants were included if both ultrasonographic ONSD measurements and contemporaneous respiratory data were available in the medical records. Newborns with major congenital malformations, known intracranial abnormalities, chromosomal disorders, or other conditions that could potentially influence intracranial pressure were excluded.

Because ONSD examinations were performed according to clinical availability rather than a predefined screening protocol, only infants with complete measurements were included in the final analysis.

The study protocol was approved by the Institutional Review Board of Ankara Bilkent City Hospital (approval date: 12 March 2020; approval number: E.Kurul-E1-19-354). The requirement for informed consent was waived because of the retrospective design of the secondary analysis.

### 2.2. Ultrasonographic Assessment of Optic Nerve Sheath Diameter

Bedside ultrasonographic examinations were performed using a high-frequency linear transducer (7–10 MHz) with a Toshiba AplioT 300 ultrasound system (Canon Medical Systems Corporation, Otawara, Tochigi, Japan). Infants were examined in the supine position using a standardized transorbital imaging technique. Minimal probe pressure was applied over the closed eyelid during image acquisition.

ONSD measurements were obtained 3 mm posterior to the globe, where the optic nerve sheath margins could be visualized most clearly. Measurements from the right and left eyes were recorded separately, and the mean bilateral value was used for statistical analysis.

All examinations were performed by the same experienced operator using predefined anatomical landmarks in an effort to reduce measurement variability during the study period. Ultrasonographic examinations were generally performed on the first postnatal day. Respiratory variables were recorded from the time point closest to image acquisition.

### 2.3. Respiratory Variables and Clinical Data

Clinical and respiratory variables were collected retrospectively from electronic medical records. Variables recorded nearest to the time of ONSD assessment included fraction of inspired oxygen (FiO_2_), peripheral oxygen saturation (SpO_2_), and the requirement for invasive respiratory support.

For subgroup analyses, infants were categorized according to respiratory support modality. Non-invasive respiratory support included nasal continuous positive airway pressure and non-invasive positive pressure ventilation. Invasive respiratory support referred to conventional mechanical ventilation delivered through endotracheal intubation.

### 2.4. Statistical Analysis

Continuous variables are presented as mean ± standard deviation or median with interquartile range according to data distribution. Categorical variables are expressed as frequencies and percentages.

Comparisons between groups were performed using parametric or non-parametric tests where appropriate. Correlations between ONSD measurements and respiratory variables were evaluated with correlation analyses.

Multivariable logistic regression analysis was performed to examine whether ONSD was independently associated with invasive mechanical ventilation. Gestational age and birth weight were included in the primary adjustment model because both variables are closely related to respiratory severity and ONSD measurements. Because the number of invasive ventilation events was relatively limited, the number of covariates in the final model was intentionally restricted to reduce the risk of overfitting.

Statistical analyses were performed using SPSS for Windows version 21 (IBM Corp., Armonk, NY, USA). A two-sided *p* value < 0.05 was considered statistically significant.

## 3. Results

### 3.1. Baseline Characteristics

A total of 110 preterm infants were included in the analysis. At the time of ultrasonographic evaluation, 40 infants (36.4%) required invasive mechanical ventilation and 70 (63.6%) received non-invasive respiratory support. ONSD measurements were generally obtained on the first postnatal day. Baseline characteristics of the study population are presented in Table 1.

Infants who required invasive mechanical ventilation had lower gestational age and birth weight compared with those receiving non-invasive respiratory support (both *p* < 0.001). Sex distribution was similar between groups (*p* = 0.55). Adequate image quality for ONSD assessment was obtained in all examinations.

### 3.2. ONSD Measurements According to Respiratory Support

**Figure 1:**
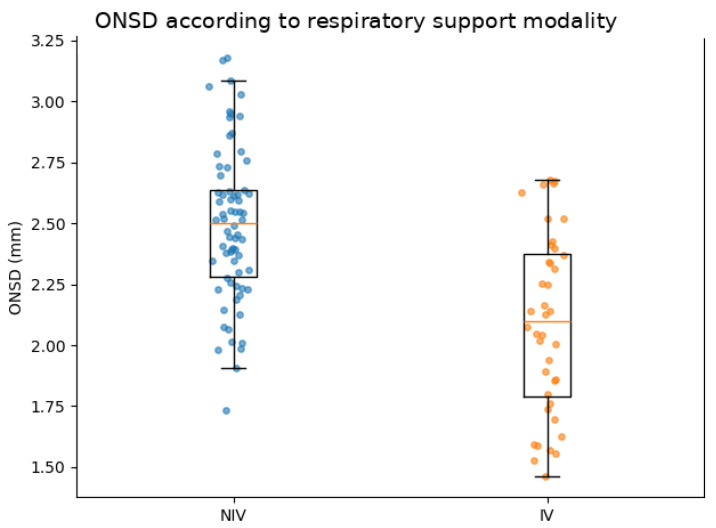
ONSD.

ONSD values were higher in infants receiving non-invasive respiratory support than in those requiring invasive mechanical ventilation (2.08 ± 0.44 mm vs. 1.60 ± 0.44 mm, *p* < 0.001; Figure 1). The absolute difference between groups was 0.48 mm.

### 3.3. Correlation Analyses

Correlation analysis demonstrated a weak negative association between ONSD and FiO_2_ that did not reach statistical significance (r = −0.16, p = 0.116) (Supplementary Figure S1). A moderate positive correlation was observed between ONSD and SpO_2_ (r = 0.30, *p* < 0.001).

In group comparisons, SpO_2_ values were higher in the non-invasive ventilation group, whereas FiO_2_ values were higher among infants requiring invasive ventilation (*p* = 0.012) (Table 2).

### 3.4. Multivariable Regression Analysis

In univariable logistic regression analysis, higher ONSD values were associated with lower odds of invasive mechanical ventilation (Table 3). After adjustment for gestational age and birth weight, however, the strength of this association decreased substantially. This finding suggests that maturational confounding accounted for much of the observed relationship between ONSD and respiratory support.

### 3.5. Sensitivity Analysis

When analyses were stratified according to gestational age, the difference in ONSD between respiratory support groups was more evident among infants born before 28 weeks’ gestation (Supplementary Table S1).

## 4. Discussion

The main finding of this study was that the apparent relationship between ONSD and respiratory support in preterm infants was largely influenced by gestational maturity. Infants who required invasive mechanical ventilation had lower ONSD values than those receiving non-invasive respiratory support, although this difference became much less pronounced after adjustment for gestational age and birth weight. Similar findings were observed in our previous study, where ONSD measurements showed strong relationships with gestational age, birth weight, and Ballard maturation scores in preterm infants [13].

Mechanical ventilation may affect intracranial dynamics through changes in intrathoracic pressure and cerebral venous return. In premature infants, however, these effects are probably small compared with the stronger influence of gestational maturity on ONSD measurements. The observed association in our cohort therefore appeared to reflect differences in maturity more than a direct physiological effect of respiratory support itself. In this population, ONSD measurements seemed to be more closely related to gestational maturity than to respiratory disease severity.

The difference in ONSD between groups was relatively small (0.48 mm) and was within the range of previously reported measurement variability in neonatal populations [14,20,21]. Earlier neonatal studies have also shown strong relationships between ONSD measurements, gestational age, birth weight, and other developmental parameters [10,11,12,13]. Similar findings have been reported in other neonatal cohorts, where ONSD values varied according to gestational maturity and anthropometric characteristics [10,11,12,13,14]. Under these conditions, separating small respiratory-related effects from the dominant influence of maturation becomes difficult. The findings suggest that gestational maturity explains much of the observed relationship between ONSD and respiratory support.

Respiratory support requirements in preterm infants are closely linked to gestational immaturity and general clinical instability. For this reason, respiratory support modality alone is unlikely to represent a specific marker of intracranial dynamics. This probably explains why the associations between ONSD and respiratory variables became weaker after adjustment for gestational age and birth weight.

These findings indicate that ONSD measurements in preterm infants should be interpreted within the clinical context of prematurity rather than used as isolated indicators of respiratory disease severity. Similar limitations have been described in the broader ONSD literature, where measurements obtained outside clearly defined intracranial hypertension states are generally interpreted together with the overall clinical picture [6,8,21].

Although bedside ultrasonography remains an attractive non-invasive tool in neonatal practice, ONSD measurements in preterm infants are influenced by several maturational and technical factors. This may limit their specificity as physiological markers [16,18,21]. In this population, ONSD measurements may reflect broader physiological characteristics related to prematurity rather than respiratory disease severity alone.

Several limitations should be acknowledged. First, the retrospective design may have introduced selection bias because ONSD measurements were performed according to clinical availability rather than a predefined protocol. In addition, ONSD examinations were performed according to clinical availability rather than systematic screening, which may have resulted in overrepresentation of infants with greater neurological or hemodynamic instability. Second, mean airway pressure, which may be one of the most relevant physiological variables linking ventilation to intracranial dynamics, was unavailable. Third, all examinations were performed by a single operator, and interobserver variability could not be assessed. This is important because the observed between-group difference in ONSD was small and within the range of previously reported measurement variability. Finally, the limited number of invasive ventilation events reduced the precision of the multivariable analyses.

## 5. Conclusions

In conclusion, ONSD measurements in preterm infants appear to be more closely related to gestational maturity than to isolated respiratory disease severity. Lower ONSD values were observed in infants requiring invasive respiratory support, but this association became weaker after adjustment for gestational age and birth weight. ONSD measurements in premature infants should be interpreted within the clinical context of prematurity rather than used alone as markers of respiratory disease severity.

## Figures and Tables

**Table 1:** Baseline characteristics of preterm infants according to respiratory support.

Variable	NIV (*n* = 70)	IV (*n* = 40)	*p*-Value
Gestational age (weeks)	29.8 ± 2.6	26.7 ± 2.9	<0.001
Birth weight (g)	1371 ± 533	919 ± 481	<0.001
Sex (male), *n* (%)	37 (52.9%)	18 (45.0%)	0.55
Apgar score at 1 min, median (IQR)	4 (3–5)	3 (2–4)	0.032
Apgar score at 5 min, median (IQR)	6 (5–7)	5 (4–6)	0.041

Continuous variables are presented as mean ± standard deviation or median (interquartile range), as appropriate.

**Table 2:** Comparison of ONSD and selected respiratory variables between ventilation groups.

Variable	NIV (*n* = 70)	IV (*n* = 40)	*p*-Value
ONSD (mm)	2.08 ± 0.44	1.60 ± 0.44	<0.001
SpO_2_ (%)	96.17 ± 2.20	94.58 ± 2.27	<0.001
FiO_2_	0.24 ± 0.04	0.28 ± 0.10	0.012

**Table 3:** Exploratory logistic regression analysis for invasive mechanical ventilation.

Variable	OR	95% CI	*p*-Value
ONSD (univariable)	0.06	0.02–0.22	<0.001
ONSD (exploratory multivariable model *)	0.03	0.002–0.74	0.032
Gestational age (weeks)	0.66	0.46–0.94	0.022
Birth weight (per 100 g)	1.32	1.00–1.74	0.053

* Exploratory multivariable model adjusted for gestational age and birth weight. Results should be interpreted cautiously because of the limited number of events and wide confidence intervals. Abbreviations: ONSD, optic nerve sheath diameter; OR, odds ratio; CI, confidence interval; NIV, non-invasive ventilation; IV, invasive ventilation.

## Data Availability

The original contributions presented in this study are included in the article/Supplementary Materials. Further inquiries can be directed to the corresponding author.

## Supplementary Materials

The following supporting information can be downloaded at: https://www.mdpi.com/article/10.3390/jcm15103732/s1, Table S1. Gestational age–stratified comparison of ONSD according to respiratory support modality; Figure S1: Relationship between fraction of inspired oxygen (FiO_2_) and optic nerve sheath diameter (ONSD).
