# Supplementary material for: Optic Nerve Sheath Diameter in Preterm Infants: Relationship with Respiratory Support and the Influence of Gestational Maturity
Source: J Clin Med. 2026 May 13;15(10):3732. doi: 10.3390/jcm15103732 (PMC13207705; doi:10.3390/jcm15103732)
Supplement: Supplementary file 1 [file jcm-15-03732-s001.zip › jcm-4283879-supplementary.pdf]

Supplementary Table S1. Gestational age–stratified comparison of ONSD according to respiratory support modality

Gestational age group	NIV (mean ONSD ± SD, mm)	IV (mean ONSD ± SD, mm)
<28 weeks	1.60 ± 0.18	1.41 ± 0.18
≥28 weeks	2.15 ± 0.42	2.09 ± 0.54

Abbreviations: ONSD, optic nerve sheath diameter; NIV, non-invasive ventilation; IV, invasive mechanical ventilation.

Relationship between FiO_2 and optic nerve sheath diameter

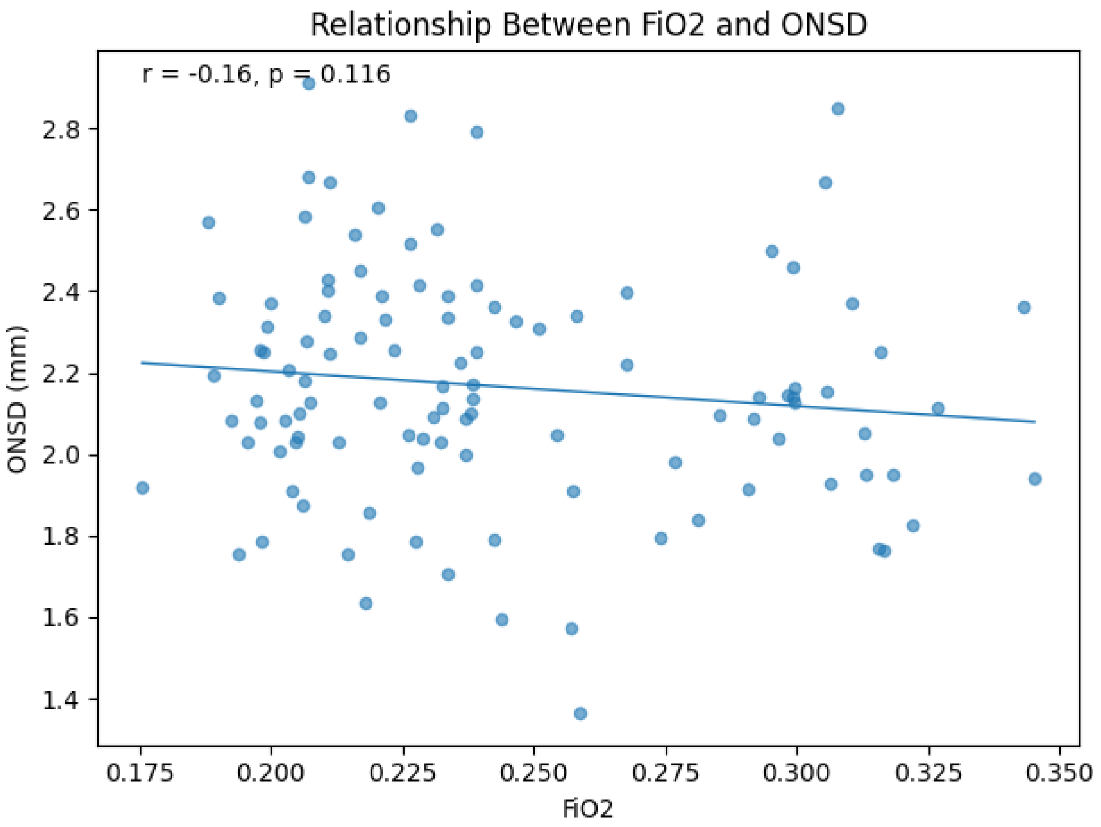

Supplementary Figure S1. Relationship between fraction of inspired oxygen (FiO_2) and optic nerve sheath diameter (ONSD). Scatter plot illustrating the association between FiO_2 and ONSD. A fitted regression line is shown for descriptive purposes. The correlation coefficient ($r = -0.16$) and p-value ($p = 0.116$) are indicated.
